# Behavioral Intervention for Adults With Autism on Distribution of Attention in Triadic Conversations: A/B-Tested Pre-Post Study

**DOI:** 10.2196/55339

**Published:** 2024-08-12

**Authors:** Onur Necip Tepencelik, Wenchuan Wei, Mirabel Luo, Pamela Cosman, Sujit Dey

**Affiliations:** 1 Electrical and Computer Engineering, University of California San Diego La Jolla, CA United States; 2 Carlsbad High School Carlsbad, CA United States

**Keywords:** autism spectrum condition, social attention, social orienting, behavioral intervention, attention distribution, triadic conversation

## Abstract

**Background:**

Cross-neurotype differences in social communication patterns contribute to high unemployment rates among adults with autism. Adults with autism can be unsuccessful in job searches or terminated from employment due to mismatches between their social attention behaviors and society’s expectations on workplace communication.

**Objective:**

We propose a behavioral intervention concerning distribution of attention in triadic (three-way) conversations. Specifically, the objective is to determine whether providing personalized feedback to each individual with autism based on an analysis of their attention distribution behavior during an initial conversation session would cause them to modify their orientation behavior in a subsequent conversation session.

**Methods:**

Our system uses an unobtrusive head orientation estimation model to track the focus of attention of each individual. Head orientation sequences from a conversation session are analyzed based on five statistical domains (eg, maximum exclusion duration and average contact duration) representing different types of attention distribution behavior. An intervention is provided to a participant if they exceeded the nonautistic average for that behavior by at least 2 SDs. The intervention uses data analysis and video modeling along with a constructive discussion about the targeted behaviors. Twenty-four individuals with autism with no intellectual disabilities participated in the study. The participants were divided into test and control groups of 12 participants each.

**Results:**

Based on their attention distribution behavior in the initial conversation session, 11 of the 12 participants in the test group received an intervention in at least one domain. Of the 11 participants who received the intervention, 10 showed improvement in at least one domain on which they received feedback. Independent *t* tests for larger test groups (*df*>15) confirmed that the group improvements are statistically significant compared with the corresponding controls (*P*<.05). Crawford-Howell *t* tests confirmed that 78% of the interventions resulted in significant improvements when compared individually against corresponding controls (*P*<.05). Additional *t* tests comparing the first conversation sessions of the test and control groups and comparing the first and second conversation sessions of the control group resulted in nonsignificant differences, pointing to the intervention being the main effect behind the behavioral changes displayed by the test group, as opposed to confounding effects or group differences.

**Conclusions:**

Our proposed behavioral intervention offers a useful framework for practicing social attention behavior in multiparty conversations that are common in social and professional settings.

## Introduction

### Background

Atypical patterns of attention in social interaction settings are a key characteristic of autism spectrum condition (ASC) [1]. Many studies of these patterns involve only two people, either as a face-to-face interaction or as a person-person-object interaction; very few analyze attention behavior in multiparty interactions. In this study, we used a head orientation estimation model to analyze the attention distribution of young adults with autism with no intellectual disabilities in triadic (three-way) conversation settings. Triadic conversations are foundational for understanding patterns in multiparty interactions as having three people in conversation is the smallest number that allows for the study of a speaker’s attention distribution across multiple listeners. In addition to the attention distribution analysis, we present a behavioral intervention framework to provide feedback on behavior relative to normative patterns. The remainder of this section includes a literature review on social attention patterns of individuals with autism and social orienting behavior in triadic interaction settings. Then, we present our experimental setting, the behavioral intervention program and results, and a discussion.

### Social Attention

Social attention is the ability and motivation to attend to a social partner during interaction, as well as coordinating attention with them [2]. Social attention includes joint attention, social orienting, eye contact, nonverbal gestures, and following social cues, such as eyes, faces, hands, and voice. Differences in social attention are a core diagnostic feature of ASC [1]. A common practice in social attention research involves presenting participants with social and nonsocial cues and analyzing their responses, enabling comparisons between different types of cues as well as populations. Many studies showed that, during face-to-face interactions, individuals with autism spend less time looking at social stimuli such as an experimenter’s face and more time looking at nonsocial stimuli such as the background or objects present in the environment than participants without autism [1-3]. The gaze behavior of children with autism and neurotypical (NT) children was compared while they watched videos that they believed to be prerecorded or live and while engaging in real social interactions [4]. The authors found that, during real social interactions, children with low autism spectrum quotient (AQ) scores looked more at the experimenter than did children with high AQ scores, while for videos, children with low AQ scores looked less at the person in the live video than in the prerecorded video, compared with children with high AQ scores. In a study by Speer et al [5], participants were presented with four types of stimuli: social dynamic, social static, isolated dynamic, and isolated static. Participants with autism exhibited significant differences under the social dynamic stimuli with decreased fixation duration on eyes and increased fixation duration on the body. Many studies on social attention patterns involve laboratory settings where social stimuli are presented digitally, such as displaying social scenes or videos on a computer screen [3,5-8]. Such laboratory interactions can lead to very different results, both qualitatively and quantitatively, compared with live interactions [9-11] in part because human eyes can communicate information as well as receive it, a dual aspect that is missing when viewing others on a screen [12]. Hence, it is important to study social communication through scenarios where a social partner is physically present [13]. Over the last decade, there has been an increase in real interaction settings to study social communication. In this study, we present a live naturalistic social interaction setting where the attention patterns of individuals with autism are examined, enabled by the unobtrusive orientation estimation system presented in our previous study [14].

Joint attention is the ability to achieve a common focus of attention with another person during a social interaction [7] and is one of the most well-studied diagnostic features of ASC [15-18]. These studies and others showed that starting from infancy, individuals with autism participate less in joint attention. More recently, Caruana et al [7] showed that adults with autism continue to have differences in joint attention, with fewer adults with autism responding to joint attention cues in their experiment, while also being slower to respond than IQ-matched controls.

Triadic interactions are significantly harder to navigate than dyadic (two-way) interactions [19]. In a dyadic interaction, objects and the background are potential distractions. A triadic interaction has those as well as an additional person who is part of the interaction and may require attention. Prior research on triadic interactions involving individuals on the autism spectrum is limited but insightful. McParland et al [20] studied triadic conversations with low communicative intent (researchers speaking primarily with each other, with occasional input from a child) and dyadic conversations with high communicative intent (a researcher directly interacting with a child). The authors found that children with autism spent 12.3% less time looking at other people’s faces in these triadic conversations than the dyadic ones, and 9.7% less than typically developing (TD) children. Authors also found that children with autism made 57% more gaze fixations to people’s faces in these triadic conversations than the dyadic ones, while TD children displayed the reverse pattern, with 61% more gaze fixations in the dyadic conversations. In our study, we aimed to create a realistic, highly communicative setting where the individual with autism is engaging with other parties continuously.

Social orienting is a rapid and involuntary attentional shift, typically by a change in gaze direction or head orientation in response to a social cue [21]. Studying the ability to follow another’s eye gaze is a common method of analyzing social orienting skills [22] and is a differentiating factor in autism [13,23-25]. Several studies analyzed head movements of people with autism [6,8,18,26,27]. Dawson et al [18] examined responses to various social cues such as hearing their own name or mother’s voice and concluded that children with autism are significantly less likely to respond to these cues with a reorientation of the head compared with TD children. Campbell et al [8] showed that fewer children with autism oriented to their names compared with TD children, and when they did respond by orienting, it took longer. Zhao et al [26] proposed a model to analyze head movement features such as rotation range and frequency in children with autism without intellectual disabilities during face-to-face interactions. The authors reported that children with autism had a significantly higher level of head movement stereotypy (repetitive, ritualistic head movements), as well as higher rotation range and frequency than TD children. Based on these results, the authors developed a machine learning model using the proposed head movement features to diagnose autism in children [27].

Many studies analyzed attention in conversational settings, including the social modulation of gaze, which is the change in gaze orientation based on conversational role (eg, speaker or listener). In dyadic conversations, listeners generally gaze more at speakers compared with speakers looking at listeners [28-31]. However, Vertegaal et al [31] found that in group conversations, the gaze levels of speakers come close to those of listeners. The authors argued that one reason for this change was that speakers, when addressing a group, need to collect visual feedback from each individual and to maintain the signal that they are addressing each individual. In a similar conversational role comparison in a face-to-face setting, it was found that when the experimenter looked directly at the participant, adults with autism looked back at the experimenter’s face less than NT adults did, missing opportunities for reciprocal social gaze [32]. Bal et al [33] showed that individuals with autism might not regularly provide normative nonverbal communication cues, such as periodically making eye contact with a speaker and maintaining a body orientation generally toward them. In a recent study [34] of social attention over longer time spans, it was found that NT individuals display an initial increase in social attention, followed by a decay and a recovery, whereas high functioning individuals with autism exhibit a constant linear decay without any recovery of attention. In a web-based public-speaking experiment [35], it was found that children with autism with no intellectual disability made contact with the listeners less frequently than TD children. In our study, the individual with autism is the primary speaker of the triadic interaction, which leads to an analysis of attention distribution for the speaking phase of a conversation.

The social attention differences discussed in this section could be attributed to reduced cognitive functioning or IQ, instead of differences in autistic traits. However, multiple studies indicated that reduced social attention in ASC is orthogonal to IQ [34]. Multiple meta-analytic studies [1,36] suggest that differences in social attention are not modulated by IQ matching between the NT and ASC groups and remain stable across IQ differences between groups [37]. Furthermore, various studies [38-40] involving control groups with developmentally delayed individuals showed that individuals with autism exhibit reduced social attention compared with both developmentally delayed and NT groups. These findings highlight the importance of addressing social attention differences in ASC, regardless of intellectual ability.

Many studies outline the impact of these kinds of social attention differences. According to Chen et al [41], differences from society’s workplace communication norms are one reason that adults with autism have high unemployment rates despite often holding college degrees, average to high IQs, and various useful skills. Furthermore, it was reported [42] that many adults were terminated from jobs due to communication differences. For the large number of individuals with autism aging into adulthood each year, effective systems that allow situational practice and feedback of social attention could support successful transition to employment. One goal of this intervention study was to provide useful feedback to adults with autism who are seeking employment.

Positive results were reported in various intervention studies involving individuals with autism. Video-based interventions were found effective in improving social communication skills [43]. Munandar et al [44] proposed a video-based intervention for college students with autism to improve their storytelling ability during job interviews, which led to positive results. Ferguson et al [45] initiated an intervention-based program for adults with autism with co-occurring intellectual disability, addressing verbal conversational skills and nonverbal behaviors such as eye contact and active listening. Promising behavioral results were reported for all pilot participants after the interventions, as well as overall positive feedback from the parents of participants. In a similar intervention program with eight sessions [46], children with autism with no intellectual disability showed significant improvements in eye contact and facial emotion recognition.

### Social Orienting in Triadic Conversations

In this study, we examine triadic conversations, a complex environment that requires keeping track of social stimuli from the other two conversational parties simultaneously and distributing attention between them. Social orienting toward a speaker is important to show interest in the conversation, and distributing attention between the other two parties while speaking can help make everyone feel included in the conversation. Joint attention is often required as well if a conversational partner initiates it toward an external point of focus.

Triadic interactions can be hard to navigate for both people with and those without autism. Auer [47] argued that one of the participants in a three-party constellation is in danger of being marginalized and may even become a bystander in extreme cases. Multiple studies [47,48] pointed out the importance of using practices such as “alternating gaze” to avoid schisms or marginalization of participants. Zima [49] argued that gaze patterns in triadic interactions are more diverse and complicated than in dyadic interactions. The author found that the gaze window pattern defined by Bavelas et al [50] occurs less frequently in triadic interactions, pointing to the difficulty of using interactive feedback in triadic storytelling activities. Arndt et al [51] showed that salespeople tend to share mutual gaze with customers who are similar to them and those who speak more; however, sharing mutual gaze equitably between customers rather than focusing on a particular customer helps with building trust and rapport. Vertegaal et al [52] argued that lack of gaze can decrease turn-taking efficiency in multiparty interactions by 25%. Many studies [53-56] showed that children were mainly excluded in pediatric visits involving triadic conversations with a parent and a pediatrician. Van Dulmen [53] argued that children could be capable of providing reliable information and feedback, and pediatricians might benefit from training to better communicate with children. These examples and others show that triadic interactions are an important setting to study both because they are common in social and professional settings and because they can act as a building block for behavior analysis of general multiparty interactions. Being able to navigate complex social interaction settings, such as triadic conversations, is important during job interviews to obtain employment or in workplaces to retain employment.

The main objective of this study is to examine the head orientations of young adults with autism during triadic conversations, before and after the receipt of a behavioral intervention. We use the privacy-preserving LiDAR-based head orientation estimation system introduced in our previous study [14]. The model works from a surveillance viewpoint at an unobtrusive distance, with sensors at two ceiling corners about 10 ft from the participants, enabling orientation analysis for real social interaction settings without the need for wearable devices or digitally created social stimuli. In our previous study [57], we showed that the orientation estimation models can reveal differences between participants with and those without autism in various parameters related to attention distribution and social orienting. In this study, we build on that and present a system that analyzes a triadic conversation session and provides personalized feedback aimed at improving various behaviors related to social attention. In a subsequent session, we study the effectiveness of the intervention.

Some of the studies presented earlier connect to this study with their focus on head movements [8,26,27], triadic interaction settings [20], or conversational settings [31-33]. However, none of these studies focus on attention distribution and head orientations in multiparty conversations. This work presents an understudied social attention setting, real triadic conversations, and presents a novel head orientation and attention distribution analysis and behavioral intervention framework regarding social communication differences in this setting.

## Methods

### Participants

Twenty-four individuals with autism, none of whom had intellectual disabilities, were involved in this intervention study. Twelve individuals with autism and eight individuals without autism with no intellectual disabilities were involved in our previous study [57], which helped design the intervention. Participants without autism were a mix of undergraduate and graduate students aged between 18 and 28 years at the time of participation. All 36 participants with autism for both the current and previous studies were recruited through a neurodiversity workforce training program organized at the University of California (UC) San Diego, which hosted two-month internship programs for employment-seeking individuals with autism every year since 2018. The interns were selected through interviews with more than 100 applicants. As per the inclusion criteria for our studies, participants with autism were at least 18 years of age and had previously received a community diagnosis of ASC. Except for a participant who was 43 years of age, the participant ages ranged from 18 to 28 years (mean 23.8 years, including the 43-year-old participant), with varying educational backgrounds. All participants were high school graduates, and most were in the process of additional education including at vocational schools, community colleges, and universities. Roughly half of the participants had some previous job experience, although it was generally short-term or part-time.

The 24 participants with autism who took part in the intervention study are split into test and control groups, with 12 participants each. The study consists of three parts for the test group: an 8- to 12-minute initial triadic conversation, an intervention, and another 8- to 12-minute triadic conversation. The participants in the control group did not receive the intervention, taking part in two consecutive 8- to 12-minute triadic conversations.

### First Conversation Session and Initial Evaluation

**Figure 1:**
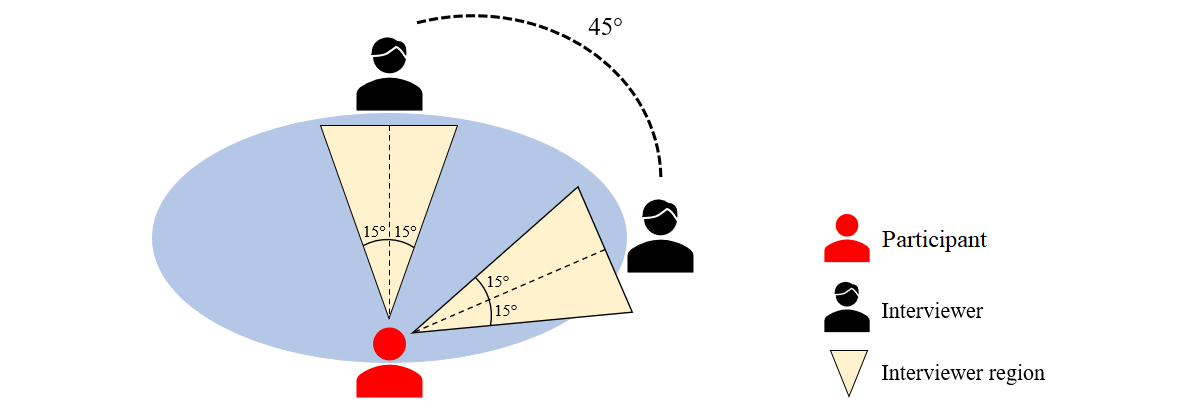
Triadic conversation setup. The participant’s head orientation is estimated continuously, and their focus of attention is analyzed using the interviewer regions depicted.

Participants converse while seated around an oval table; one interviewer is across from the participant and the other is seated to the side, so a head turn by the participant from one interviewer to the other corresponds to about 45 degrees as shown in Figure 1. The conversation is conducted in a semistructured interview style [58], which starts with the following question: “What do you do in your free time?” and continues based on the answers of the participant. The interviewers are instructed to make sure that the participant is the main speaker throughout the conversation, while they listen in an engaged way, make brief comments, ask follow-up questions, and shift topics when necessary.

Throughout the conversation, the participant’s head orientation is estimated and recorded as a sequence using the model from our previous study [14]. Multiple studies [52,59,60] showed that head orientation is a good indicator of visual focus of attention, without the need to estimate gaze orientation. To analyze the participant’s attention distribution, we define two events based on their duration, *contact* and *exclusion*. Although there are studies of gaze patterns or orientation behaviors in triadic interactions, none define specific communication events pertaining to excluding participants and instead rely on qualitative analyses. Multiple studies [47,48] discussed the danger of exclusion in triadic interactions but did not specify how long it takes for someone to be considered excluded. We use the definitions of these events from our previous study [57]; the participant makes *contact* with an interviewer if their head orientation corresponds to that interviewer’s region (spanning 15 degrees to either side of the interviewer; see Figure 1) for three consecutive frames (about 2.5 seconds, given the frame rate of about 1.3 fps). Similarly, an *exclusion* happens in a 20-frame window where at least 15 frames fall in the region of one interviewer and none in the region of the other. To estimate the head orientations, we developed a neural network regression model that uses handcrafted geometric features extracted from human point clouds. The estimated head orientation sequence is analyzed continuously on a rolling basis; a *contact* or an *exclusion* is initiated as soon as one of the required conditions happens and is terminated as soon as the condition no longer holds. As presented in our previous study [57], these two events capture important aspects of social communication and can characterize distinct patterns of attention for individuals with and those without autism, covering two important parameters of gaze, duration, and frequency, as defined by Kleinke [30]. Table 1 shows the five domains for which the head orientation sequence is analyzed, the averages for these according to data from 8 participants without autism and 12 participants with autism, as well as the SD among the participants without autism and the values corresponding to 2 SDs from the mean [57]. We also showed in our previous study [57] that some of the behavioral differences between the groups with and those without autism shown in Table 1 are statistically significant.

The duration, pace, topics, and conversational roles were kept very similar to prevent these external factors from confounding the results. Each participant was asked questions about the same set of topics in the same order. The interviewers asked only short questions and made brief comments when necessary, making the participant the main speaker of the conversation. In all 48 conversation sessions, the participant was the speaker 81%-93% of the time, disregarding the back channels and considering only those sequences where a speaker is talking for at least 2 seconds.

**Table 1:** Statistical domains for attention-related behaviors for which the interventions are provided if a participant exceeds nonautistic average ±2 SDs. Numbers are in terms of frames for *AvgCon*, *AvgNoCon* and *MaxExc*: 1 frame≈0.75 seconds. Em dashes (—) represent directions for which no intervention was given.

Statistic	NA^a^ average (SD)	ASC^b^ average	NA average + 2 SDs	NA average – 2 SDs
Average duration of contact (*AvgCon*)	6.09 (0.94)	7.73	7.97	4.21
Average duration of NOT making contact with anyone (*AvgNoCon*)	30.95 (15.11)	58.6	61.17	—
Number of contacts per minute (*NumCon*)	2.14 (0.47)	1.44	—	1.21
Maximum exclusion duration (*MaxExc*)	9.38 (5.89)	20.3	21.16	—
Total duration of exclusion percentage per session (*ExcPct*)	3.13% (1.85%)	7.4%	6.83%	—

^a^NA: nonautistic.

^b^ASC: autism spectrum condition.

### Behavioral Intervention

The intervention session was conducted by a researcher who was trained by a behavioral coach. The discussion starts with the researcher explaining the experimental setup and its purpose, including information on the importance of distributing attention in triadic interactions, using body and head orientations to facilitate engagement, and our orientation estimation models and tools to analyze attention distribution. The researcher also provides an example of an unwanted situation, where two parties of a triadic conversation orient directly toward each other and exclude the third person from the interaction. The researcher encourages the participant to reflect on their session, with questions such as “Do you think you made any social exclusions?” This part of the intervention is orchestrated identically for all participants in the test group.

Based on the head orientation analysis for the first conversation session, for a given domain, additional intervention (data analysis and video examples) is provided to participants in the test group if they deviate from the NA mean by at least 2 SDs. We found in our previous study [57] that, on the five metrics shown in Table 1, the average value for the participants with autism differed from the average NA score by about 2 SDs. Note that for most metrics, deviations to only one side of the NA average are considered for intervention, since making more contacts per minute or making no exclusions at all is not a behavior that would suggest feedback. Each participant was provided feedback on up to three domains. If a participant exceeded the threshold of 2 SDs on more than three domains, the domains where the deviations are the largest were selected for the intervention.

Next, the data analysis from the initial session is shared with the participant, focusing on up to three domains where the participant deviated from the NA average by more than 2 SDs. The participant is informed about the NA average and by how much they deviated from the NA average. The researcher helps the participant think about their attention distribution patterns during the first session with questions such as: “Why do you think your average contact duration was high?” or “Did you think you excluded one of the listeners?” Finally, participants are shown examples of attention distribution videos, taken from triadic conversation sessions involving participants without autism. Each domain has a different associated video that demonstrates the social attention aspect on which a participant is receiving intervention. While the participant watches the video, the researcher asks additional open-ended questions such as: “What do you notice about the participant’s head orientations in the video?” Each participant who received an intervention completed the same steps of discussion, data analysis, and video example detailed in this section. In total, the intervention session took 10-15 minutes. For participants in the control group, no intervention is given; there was merely a short break between the two conversation sessions.

### Second Conversation Session

Participants took part in a second conversation session, similar to the first. Upon completion of the second conversation session, the researcher asked the test participant about how it felt to do a second session after the intervention, and whether they did anything differently. Finally, all participants were presented with the option to review and discuss their statistics and head orientation analysis.

### Ethical Considerations

This study was approved by the UC San Diego Institutional Review Board (Protocol 210775; July 1, 2021). Participants gave their informed consent to the research team to analyze their individual data by signing a consent form describing the study and the data collection procedure. The privacy of the participants is protected through the assignment of deidentifying codes to each participant throughout this manuscript. Participants received US $15 for an hour of participation as compensation.

## Results

**Figure 2:**
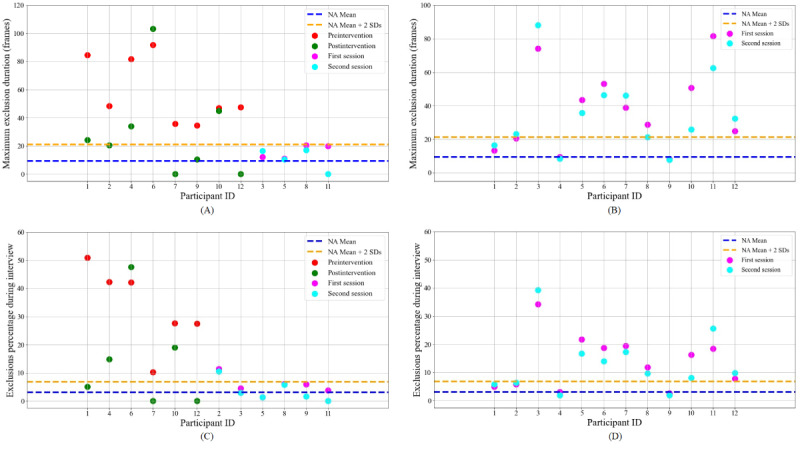
Participant data for exclusion-related behaviors in first and second conversation sessions for test and control groups. (A) Maximum exclusion duration (*MaxExc*) statistics for the test group. (B) *MaxExc* statistics for the control group. (C) Exclusions percentage (*ExcPct*) statistics for the test group. (D) *ExcPct* statistics for the control group. NA: nonautistic.

**Figure 3:**
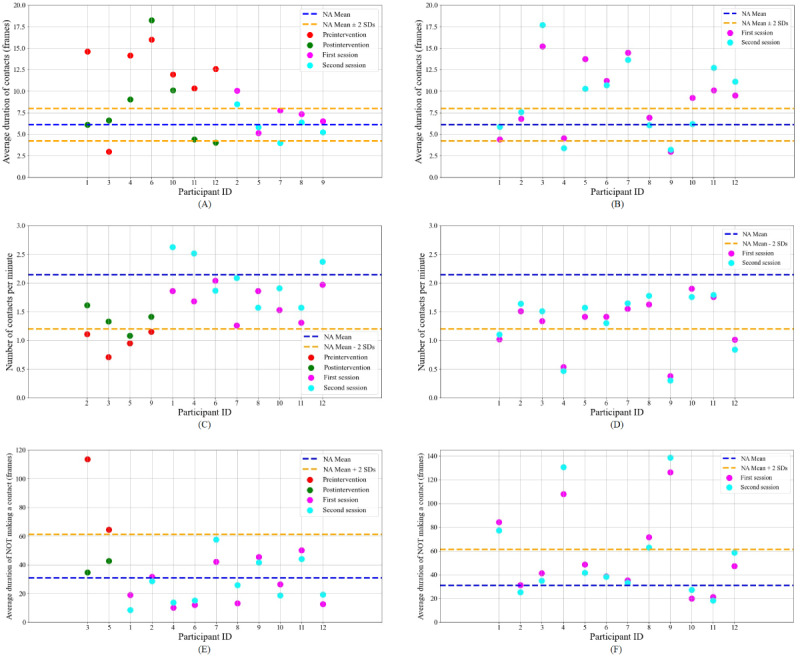
Participant data for contact-related behaviors in first and second conversation sessions for test and control groups. (A) Average duration of contact (*AvgCon*) statistics for the test group. (B) *AvgCon* statistics for the control group. (C) Number of contacts per minute (*NumCon*) statistics for the test group. (D) *NumCon* statistics for the control group. (E) Average duration of not making contact with anyone (*AvgNoCon*) statistics for the test group. (F) *AvgNoCon* statistics for the control group. NA: nonautistic.

Our chosen thresholds meant that 11 of 12 test participants received intervention on at least one domain, with most participants receiving interventions on two or three domains. Participant 8 did not receive an intervention as their behavior was consistent with NT norms for all five domains. The most common domains for intervention were related to *exclusions*, “maximum exclusion duration” (*MaxExc*) and “total duration of exclusion percentage per session” (*ExcPct*), with the thresholds suggesting feedback for 8 and 6 participants, respectively. For *contact*-related statistics, 6 participants received intervention on high “average duration of contact” (*AvgCon*) and 1 participant received intervention on low *AvgCon*, 4 participants received intervention on “number of contacts per minute” (*NumCon*), and 2 participants received intervention on “average duration of not making contact with anyone” (*AvgNoCon*). Figure 2 shows the data for *exclusion*-related domains, in comparison with the NA averages and the intervention region (2 SDs from the NA mean). Figures 2A and 2C show data for the test participants. Those participants receiving intervention for that domain (left side of plot) are marked with red and green dots, corresponding to their scores during the first and second conversation sessions. The test participants who did not receive intervention for that domain (right side of plot) are marked with magenta and cyan dots for their first and second sessions, as also is the case in Figures 2B and 2D, showing the control group. Figure 3 shows the data for *Contact* related statistics. We compared the performances in the first conversation session for the test and control groups to check for between-group differences; no significant difference was observed in any domain (*P>.*10 for all five domains).

Figure 2A shows the results for *MaxExc*. Eight participants exhibited a maximum exclusion duration higher than the NA average by at least 2 SDs in their first session, meaning that one of the interviewers was excluded from the conversation for a long period of time, at least once. Hence, these participants received intervention about this behavior. According to an independent 1-tailed *t* test, the changes in *MaxExc* for the group of 8 participants who received the intervention are statistically significant compared with the control group, t_18_=−3*.*3; *P*=*.*004. To assess individual improvements, we use 1-tailed Crawford-Howell *t* tests [61-63], which allow for single case comparisons against a control group. This test accounts for the small size of the control group and the uncertainties over its mean and SD [62]. According to 1-tailed Crawford-Howell *t* tests, 6 participants out of the 8 who received the intervention made statistically significant improvements in *MaxExc* compared with the control group (exceptions are participants 6 and 10).

Figure 2C shows the results for *ExcPct*. All participants apart from participant 6 made statistically significant improvements according to 1-tailed Crawford-Howell *t* tests. As a group, the 6 participants who received the intervention made statistically significant improvements in *ExcPct* compared with the control group, t_16_=−3.4; *P*=.004.

Results are generally similar for the contact statistics. Figure 3A shows results for *AvgCon* for which 6 participants received intervention due to a high *AvgCon* and 1 received intervention due to a low *AvgCon*. As a group they made significant improvements and all but 2 (participants 6 and 10) improved compared with the control group. Similarly, Figure 3C presents results for *NumCon*, where 4 participants received intervention for having a low *NumCon*. We drop the independent *t* test for smaller intervention groups, but 1-tailed Crawford-Howell *t* tests confirm that 3 participants out of the 4 who received the intervention made significant improvements in *NumCon*, compared with the control group. Only 2 participants received intervention on *AvgNoCon*, both of whom improved according to 1-tailed Crawford-Howell *t* tests compared with the control group, as displayed in Figure 3E.

**Figure 4:**
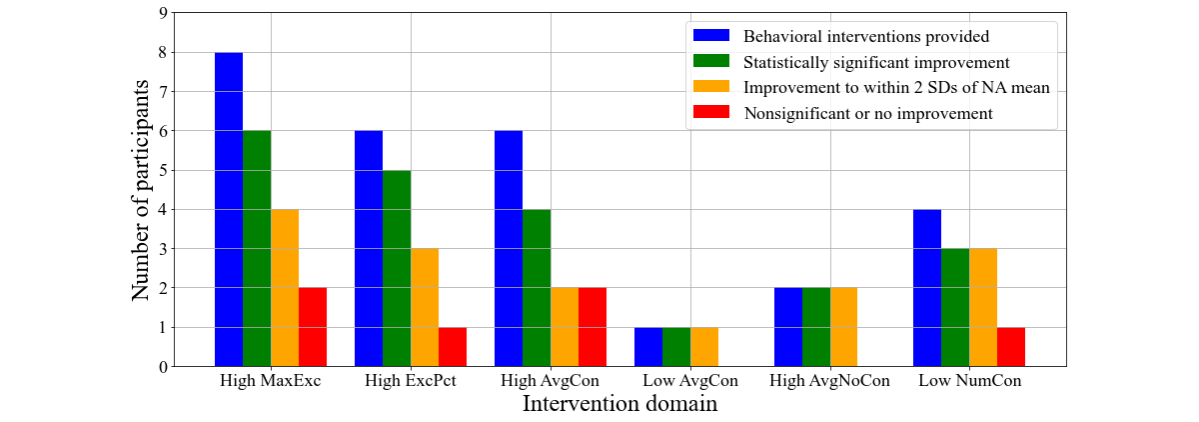
Cumulative statistics of interventions provided to participants. Interventions were provided on 27 statistical domains and 21 of those resulted in statistically significant improvements. NA: nonautistic.

Figure 4 displays cumulative results. In total, our model proposed an intervention on 27 domains, and 21 (78%) of these intervention cases resulted in statistically significant improvements in the second conversation session compared with the control group. For 15 intervention cases (56%), the participants not only showed significant improvements but also moved to the no-intervention region. Of the 11 participants who received intervention, 10 showed significant improvement in at least one domain on which they received feedback.

We conducted statistical tests on the control group to check whether external factors such as familiarity with the interviewers or the conversation setting might have had a significant effect. For all five domains, the changes between the first and second conversation sessions are nonsignificant for the control group (*P>.*10 for all five domains).

## Discussion

### Principal Findings

In this study, we propose a behavioral intervention based on a social attention analysis in triadic conversations using the estimated head orientations of the participants. In our previous study [57], differences in attention distribution between individuals with and those without autism were analyzed, and metrics related to *exclusions* and *contact* were quantified for the 2 groups. Using these metrics, we designed a study where participants receive personalized feedback on their attention patterns. Twelve participants on the autism spectrum formed the test group, of whom 11 received interventions on specific domains and 10 showed improvement in the postintervention session. The numerical results in the postintervention session suggest that the intervention session was effective compared with the control group of 12 individuals with autism who did not receive an intervention.

In this study, the intervention session was conducted by the researcher who was trained by a behavioral coach. As the first iteration of this study, having the researcher present during the intervention session enabled us to answer the questions of the participants, observe their reactions, and potentially refine the approach. We concluded that the information presented during the intervention discussion is clearly understandable by the participants. Poststudy discussions with intervention recipients revealed that 4 participants found the discussion with the researcher to be the most informative and impactful way of delivering the intervention, while 3 preferred the data analysis part and 1 participant found the video example the most useful. These results suggest that providing feedback in multiple ways can be helpful and different people may prefer different forms of feedback.

The results of this study suggest that an intervention on orientation behavior may enable individuals with autism to modify their attention behavior to become more similar to NT norms of social attention. Such adjustments could, in turn, help these individuals obtain and retain employment. As more companies move in the direction of making their interview practices and workplace environments friendly for applicants with autism and employees, the need for individuals with autism to adjust their own social communication patterns may decline. For the foreseeable future, however, some adjustment to fit in with the social communication norms of workplaces is likely to remain important for people on the autism spectrum to find and retain employment [41,42]. Many intervention studies [43-46] were proposed and achieved positive results in helping individuals with autism make those adjustments, focusing on different aspects of social attention and targeting different age groups. In this study, we focus on attention distribution and orientation behavior during triadic interactions, which is an understudied experimental setting. Our study is designed for adults with autism with no intellectual disabilities, which is a relatively undertargeted population compared with children or individuals with autism with intellectual disabilities. To the best of our knowledge, this intervention study is the first to use this experimental setting and target this population.

### Limitations and Future Work

The main limitation of this study is the small number of participants involved. The thresholds for determining when to provide an intervention were created using the data from 8 participants without autism, while the main study involved 24 participants with autism. The Crawford-Howell *t* test accounts for the small sample size of the control group.

The intervention sessions during this study were conducted with in-person feedback. In future iterations of this work, we aim to provide this feedback in a fully automated way, so that the method can be easily replicated and users can use the proposed models as self-practice tools. We will also address the low number of participants.

### Conclusions

We proposed a triadic conversation study to provide a behavioral intervention on social attention patterns to individuals on the autism spectrum. This triadic conversation study was successful in providing feedback to participants both objectively and subjectively. Comparison between the first session and the postintervention session shows clear improvements, confirmed by significance tests in comparison with the control group. The proposed triadic conversation setup and feedback model can enable situational practice for triadic interactions, which are common in both social and professional settings.

## Abbreviations

AQ: autism spectrum quotient
ASC: autism spectrum condition
AvgCon: average duration of contact
AvgNoCon: average duration of not making contact with anyone
ExcPct: total duration of exclusion percentage per session
MaxExc: maximum exclusion duration
NA: nonautistic
NT: neurotypical
NumCon: number of contacts per minute
TD: typically developing
UC: University of California

## Data Availability

The data sets generated and analyzed during this study are not publicly available due to privacy restrictions.
